# Object Distance Estimation Using a Single Image Taken from a Moving Rolling Shutter Camera

**DOI:** 10.3390/s20143860

**Published:** 2020-07-10

**Authors:** Namhoon Kim, Junsu Bae, Cheolhwan Kim, Soyeon Park, Hong-Gyoo Sohn

**Affiliations:** School of Civil and Environmental Engineering, Yonsei University, Seodaemun-gu, Seoul 03722, Korea; kim_namhoon@yonsei.ac.kr (N.K.); junsu510@yonsei.ac.kr (J.B.); pat5789@yonsei.ac.kr (C.K.); sypark93@yonsei.ac.kr (S.P.)

**Keywords:** rolling shutter camera, rolling shutter effect, jello effect, single photo, distance estimation

## Abstract

This paper proposes a technique to estimate the distance between an object and a rolling shutter camera using a single image. The implementation of this technique uses the principle of the rolling shutter effect (RSE), a distortion within the rolling-shutter-type camera. The proposed technique has a mathematical strength compared to other single photo-based distance estimation methods that do not consider the geometric arrangement. The relationship between the distance and RSE angle was derived using the camera parameters (focal length, shutter speed, image size, etc.). Mathematical equations were derived for three different scenarios. The mathematical model was verified through experiments using a Nikon D750 and Nikkor 50 mm lens mounted on a car with varying speeds, object distances, and camera parameters. The results show that the mathematical model provides an accurate distance estimation of an object. The distance estimation error using the RSE due to the change in speed remained stable at approximately 10 cm. However, when the distance between the object and camera was more than 10 m, the estimated distance was sensitive to the RSE and the error increased dramatically.

## 1. Introduction

Currently, most of the cameras used by the general public are mounted on, for example, smartphones, digital single-lens reflex (DSLR) cameras, and mirrorless cameras with rolling shutters. Rolling shutter refers to a method in which the entire sensor pixel does not acquire the values at once, but sequentially obtains the values. Reasons for using this shutter type include reductions in the manufacturing cost of the sensor, power consumption, and data processing costs [1]. With its many advantages, rolling shutter cameras are used in various technologies, such as robots, drones, handheld cameras, and smartphones.

Due to the characteristics of the sensor that acquires the pixel values sequentially starting from the top row of the sensor, a specific phenomenon called the rolling shutter effect (RSE) or jello effect appears in the image captured with a rolling-shutter-type camera. In the RSE-expressed image, the object is tilted, the length is changed, or the shape is deformed. A rolling-shutter-type sensor is an unfamiliar concept, but some research is being conducted. Forssen and Ringaby [1] mathematically modeled the motion of a handheld video camera to perform RSE correction. Their work has shown that camera movement can be most effectively corrected when modeled using a rotation-only model. Meingast et al. [2] studied a geometric model of a rolling shutter camera. The authors not only established a geometric model according to the movement of the camera but also studied the calibration of the rolling shutter camera. They emphasized that the modeling for the rolling shutter camera must be considered because these camera types are widely used in dynamic systems, such as unmanned aerial vehicles (UAVs) and ground robots. Yoon et al. [3] conducted a study to identify the structural system using a rolling shutter mounted in the UAV and proposed a technique to correct the rolling shutter effect in this process.

In the literature [4,5], the movement path of the camera was interpolated using a spline. Based on the proposed mathematical model of the rolling shutter, Ait-Aider et al. [6] estimated the pose and speed of a moving object using a single photo. In their study, a method for simultaneously calculating the pose and instantaneous velocity of an object was proposed. Other research includes accurately obtaining three-dimensional positions of the objects using multiple images from a rolling shutter camera [7,8,9,10,11]. Ait-Aider and Berry studied the use of a rolling shutter camera projection model for a spatiotemporal triangulation of rolling shutter stereo images [7].

A bundle adjustment was performed using a rolling-shutter-type smartphone and a compact camera [8,9]. Kim et al. [10] applied the rolling shutter concept to simultaneous localization and mapping (SLAM) photogrammetry and Vautherin et al. [11] to UAV photogrammetry to improve the three-dimensional positional accuracy. Zhou et al. [12] performed mathematical modeling of the sensor’s readout time to eliminate distortion caused by the rolling shutter and performed bundle block adjustment. The discussions have been made on the relative pose estimation [13] and absolute pose estimation [14,15] of the rolling shutter cameras. Schubert et al. [16] developed a visual-inertial odometry system that combines the inertial measurement unit (IMU) measurements with the pose and velocity estimates using the geometry of the rolling shutter. Taketomi et al. [17] highlighted the importance of applying the rolling shutter model in visual SLAM. The calibration of rolling shutter sensors has also been addressed as an important research topic [2,18,19,20]. Lee et al. [21] tried to fuse sensors of a low-cost IMU and a rolling shutter camera. For this, the authors presented a technique to estimate the calibration and noise parameters of a low-cost IMU and rolling shutter camera. In summary, it is necessary to apply a particular mathematical model for rolling-shutter-type cameras as the object or camera moves while the photograph is taken, and many studies can be conducted using the distortion caused by the characteristics of the rolling shutter. However, most studies mainly focused on estimating the exterior orientation parameters (EOPs) and deriving an accurate geometrical model of rolling shutter cameras.

Although many works are concentrated on correcting the RSE or modeling the EOPs, there is little research on obtaining geoinformation from the RSE occurrence. Based on our observation on the images taken from a rolling shutter camera, the extent of the RSE varies depending on the speed of the camera or the object and distance between the camera and the object. We had an idea to uncover the distance relationship between a camera and an object using a single image obtained with a rolling-shutter-type camera.

Some attempts have been made to obtain three-dimensional information from a single image. Chen et al. [22] reconstructed a three-dimensional model by combining the cognitive ability of humans with the computational accuracy of computers using a single image. The authors provided a modeling gesture-based tool, the so-called 3-sweep, as general objects can be divided into cylinders, cubes, etc. In this study, the authors proposed a method for reconstructing a three-dimensional shape by combining human cognitive ability and computational accuracy of a computer. This method has a strong performance in recreating a three-dimensional shape, but it does not provide a solution for determining the three-dimensional position of an accurate object, and there is a limitation in reproducing a shape like a natural object rather than an artificial one. Other studies [23,24] reproduced three-dimensional shapes using image processing and the structural characteristics of objects. Since these studies extract 3D shapes from a single image using existing 3D models, they show strong results for objects with 3D models but do not show good results for objects that do not. Besides, there is a disadvantage in that the scale parameter cannot be specified. The methods cannot extract three-dimensional information as the geometrical arrangement of cameras and objects were not mathematically provided.

While conducting experiments by investigating literature on the rolling shutter camera, we found that the distance between the camera and the object increased and the size of the RSE appeared differently. Many papers have shown that the size of the RSE is related to the shutter speed of the camera or the speed of movement of the camera or object, but has not reported that it is related to the distance between the camera and the object. Based on intensive reviews of rolling shutter cameras, the aim of this study is to measure the distance to an object using a rolling shutter camera attached to indoor robotics, a mobile mapping system (MMS), and other ground moving platforms. The relationship between the camera and an object was geometrically modeled by assuming three scenarios; movement parallel to the image plane, movement perpendicular to the image plane, and movement at a certain angle to the image plane. In this paper, the variables affecting the RSE are listed and mathematically organized. The degree of the RSE was mathematically modeled using these variables, and the vertical distance between the object and the camera was derived using the RSE. Finally, the actual distance between the object and the camera was obtained using the geometry of the camera sensor. This process was performed using the geometric arrangement of the object, camera, and camera sensor. Through this study, we aimed to develop equations for estimating distance using low-cost optical equipment. In addition, this study was conducted to investigate the possibility of obtaining simple three-dimensional information without using expensive equipment such as light detection and ranging (LiDAR) or radar devices.

The paper is arranged as follows: In the next section, an overview of the rolling shutter and a brief explanation of the RSE is provided, and the distance between the camera and the object is derived using the degree of the RSE. Subsequently, details are given on the experiment for verifying the equations presented in this paper using the test data. Finally, the conclusions of this study are presented.

## 2. Distance Estimation Using Rolling Shutter Effect

### 2.1. Rolling Shutter Camera and Rolling Shutter Effect (RSE)

**Figure 1:**
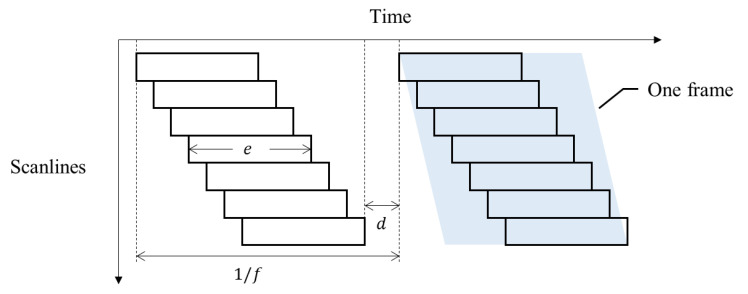
Rolling shutter camera exposure timeline.

Rolling shutter is a type of shutter that works sequentially. The camera sensor pixels of the rolling shutter are exposed downward consecutively from the top row [7]. Due to the characteristic of the rolling shutter, images differ from those taken using a global shutter. The conventional film cameras or surveying cameras use global shutters, where the shutter closes at the same moment and every pixel of the charge-coupled device or complementary metal-oxide-semiconductor acquires a value simultaneously. For manufacturing and price advantages, producers often use rolling shutters when manufacturing cameras. Figure 1 shows how the rolling shutter works. The frame rate (*f* fps), exposure of one row (*e* s), and delay between frames (*d* s) are the variables of a rolling shutter camera [2].

**Figure 2:**
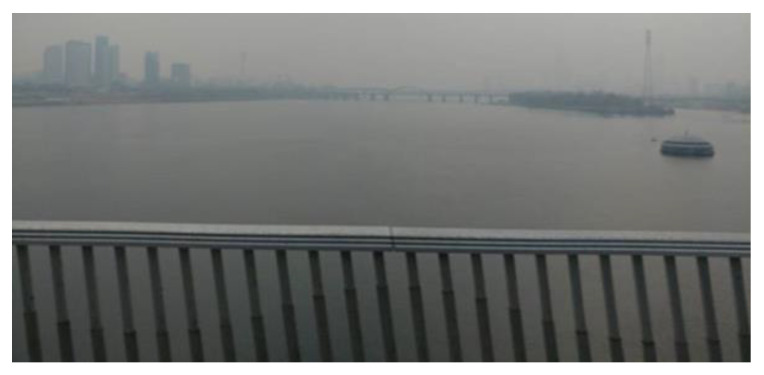
Example of the rolling shutter effect (RSE).

Due to the operational sequence of a rolling shutter that sequentially assigns values for each row pixel, geometric distortion occurs in the captured image frame. When photographing moving objects with rolling shutter cameras, such as automobiles, electric fans, strings of string instruments, etc., distorted shapes are captured. This is because the object moves or rotates while the shutter opens and closes. In general, it is known that the extent of the RSE depends on the speed of the object and the shutter speed of the rolling shutter camera [11,25]. Figure 2 shows an example of the RSE. The vertical railing of the bridge is slightly tilted due to the RSE.

The RSE generated in the image causes various issues. First, a linear object may be inclined or occur in a curved shape due to RSE, which may affect the image processing technique. When photographing a rotating object, the shape of the object itself may appear bizarrely distorted. From a photogrammetric point of view, when a global shutter model is applied to a rolling shutter camera, an error occurs because the camera’s EOP is not properly reflected.

### 2.2. Derivation of Distance Estimation Equation Using the RSE

**Figure 3:**
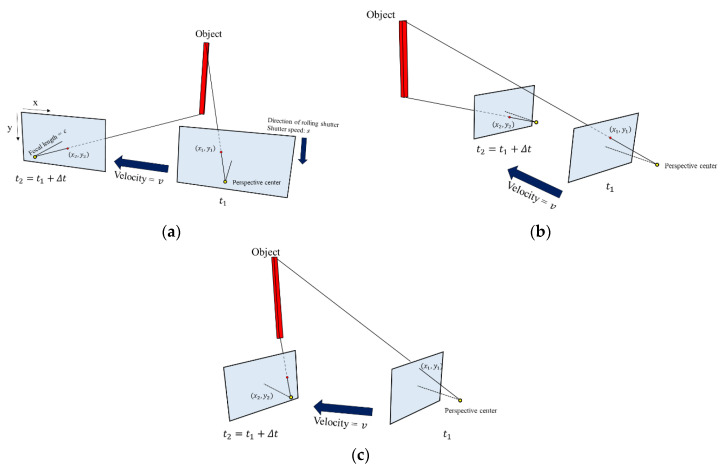
Camera moving scenario: (**a**) parallel to the image plane; (**b**) perpendicular to the image plane; and (**c**) at a certain angle to the image plane.

Figure 3 shows photographing scenarios using a rolling-shutter-type camera with a focal length *c*, pixel size *p*, principal point pixel coordinates *p_x_*, *p_y_*, and shutter speed *s*, which is moving at speed *v*. Let *t*_1_ be the time when the top of the object standing vertically is captured, and *t*_2_ be the time when the bottom end is captured. The pixel coordinates where the top of the object is photographed due to the RSE are (*x*_1_, *y*_1_) and the pixel coordinates where the bottom is photographed are (*x*_2_, *y*_2_).

**Figure 4:**
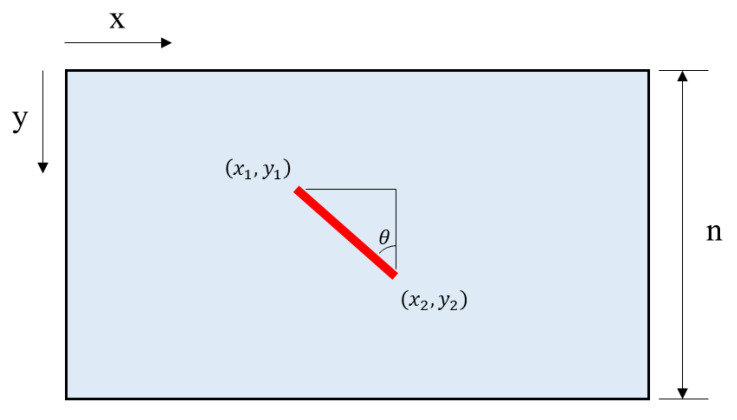
Image parameters and the RSE.

The final image looks like that in Figure 4. It can be seen that the object was photographed slantingly due to the RSE. Let θ be the degree of RSE in the image with row number *n*. This phenomenon can occur when either the camera or the object moves.

#### 2.2.1. Derivation of the RSE Equation: The Movement of the Object Parallel to the Image Plane

The shutter speed *s* is the time taken to shoot from the top row to the bottom row. Assuming that the shooting time of each row is the same, the time taken to shoot one row is *s/n* seconds, and the number of rows taken during time Δ*t* is *n*Δ*t/s*. Therefore, the difference of *y* coordinates caused by the RSE is shown in Equation (1).

(1) $$y_{2}=y_{1}+\frac{n}{s}\Delta t$$

**Figure 5:**
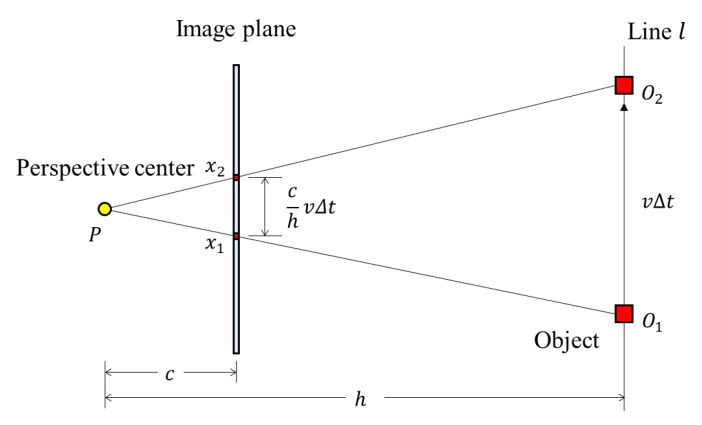
The RSE occurrence with the camera parameters and the moving scenario parameters (object moves sideways).

In Figure 5, the distance between pixels *x*_1_ and *x*_2_ can be induced by Δ*PO*_1_*O*_2_ and Δ*Px*_1_*x*_2_. Let vertical distance *h* be the distance between the perspective center of the camera and the line *l*, which connects points *O*_1_ and *O*_2_. The distance between the positions of pixel coordinates *x*_1_ and *x*_2_ can be calculated by the similarity of triangles. Equation (2) is the distance relationship between *x*_1_ and *x*_2_. Equation (3) is the relationship between the RSE and variables, and Equation (4) is the vertical distance from the perspective point to the object.

(2) $$x_{2}=x_{1}+\frac{cv\Delta t}{ph}$$

(3) $$\tan\theta =\frac{x_{2}-x_{1}}{y_{2}-y_{1}}=\frac{\frac{cv\Delta t}{ph}}{\frac{n\Delta t}{s}}=\frac{cvs}{nph}=\frac{\left(focal\;length\right)\times \left(velocity\right)\times \left(shutter\;speed\right)}{\left(number\;of\;rows\right)\times \left(pixel\;size\right)\times \left(vertical\;distance\right)}$$

(4) $$vertical\;distance=\frac{\left(focal\;length\right)\times \left(velocity\right)\times \left(shutter\;speed\right)}{\left(number\;of\;rows\right)\times \left(pixel\;size\right)\times \left(\tan\theta \right)}$$

**Figure 6:**
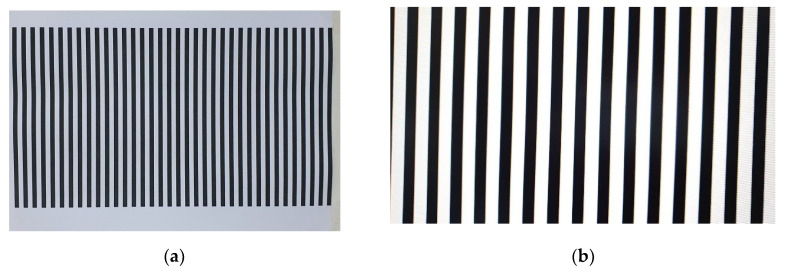
RSE occurrence test result: (**a**) the test site and (**b**) the image taken when the camera moves parallel to the image plane.

From Equation (3), if the vertical distance between the camera and object remains constant, the amount of RSE becomes also constant over the image. We verified this using an indoor test. After attaching an image of vertical lines on the wall, the camera moved parallel to the wall. Figure 6a is a test image of vertical lines attached to the wall, and Figure 6b is the resulting image after the camera moving parallel to the wall. As can be seen from Figure 6b, the RSE occurred in the same direction and the same amount in all regions of the image.

**Figure 7:**
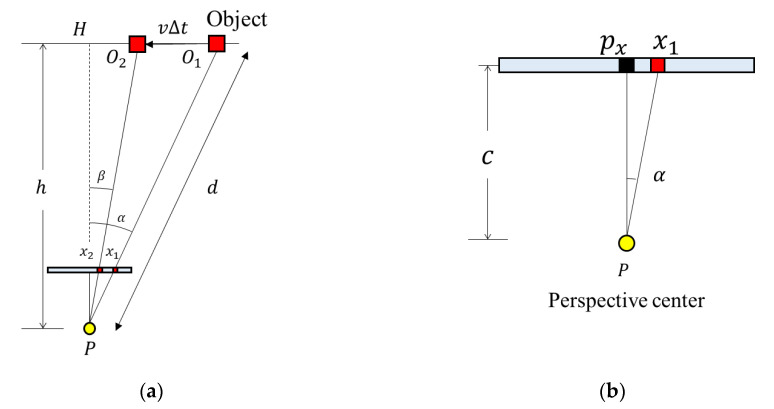
Bird’s-eye view of the RSE occurrence: (**a**) relationship between the vertical distance and the distance and (**b**) angle between the pixel, the perspective center, and the principal point.

This formula can be applied when the RSE occurs in the center of the image (*h* is approximately equal to *d*, *h* ≈ *d*). The following Figure 7 presents a scenario when the RSE occurs at the side of the image and can be applied when *h* and *d* do not have similar values. Let the foot of the perpendicular from *P* on the line *l* be *H*, and let ∠*HPO*_1_, ∠*HPO*_2_ be α, β (Figure 7a). In general, it can be assumed that α≈β because Δ*t* is sufficiently small and the length of the line $\bar{O_{1}O_{2}}$ is sufficiently small compared to *h*.

Equation (5) is the distance from the camera to the object. In Figure 7b, α is the angle between the principal point, the perspective center, and the image point. Equation (6) shows the relationship among the pixel coordinate of the principal point *p_x_*, the pixel size is *p,* and the focal length is *c*. Therefore, the distance between the camera and the object can be expressed as Equation (7).

(5) $$d=\frac{h}{\cos\alpha }$$

(6) $$\tan\alpha =\frac{\left|\left(x_{1}-p_{x}\right)p\right|}{c},\;\alpha =\arctan\left(\frac{\left|\left(x_{1}-p_{x}\right)p\right|}{c}\right)$$

(7) $$\begin{array}{ll} distance & =\frac{vertical\;distance}{\cos\alpha }\\ & =\frac{vertical\;distance}{\cos\left[\left(\arctan\left(\frac{\left|\left(x_{1}-p_{x}\right)p\right|}{c}\right)\right)\right]}\\ & =\frac{\left(focal\;length\right)\times \left(velocity\right)\times \left(shutter\;speed\right)}{\left(number\;of\;rows\right)\times \left(pixel\;size\right)\times \tan\theta \times \cos\left[\left(\arctan\left(\frac{\left|\left(x_{1}-p_{x}\right)p\right|}{c}\right)\right)\right]} \end{array}$$

As the RSE is generated when the camera moves at a speed of *v* is the same as that generated when the object moves at the speed of *v*, the distance formula can also be derived in the same way.

#### 2.2.2. Derivation of the RSE Equation: The Movement of the Object is Perpendicular to the Image Plane

**Figure 8:**
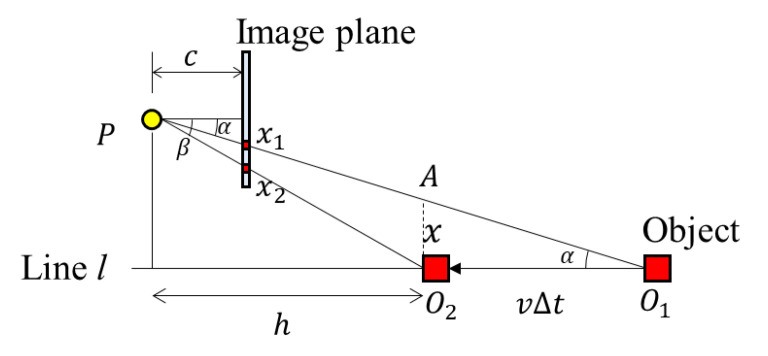
The RSE occurrence with the camera parameters and the moving scenario parameters (object moves back and forth).

When the camera or object moves back and forth, the conditions are the same as when the camera or object moves sideways. Therefore, the difference of the *y* coordinates caused by the RSE is the same as that when the object moves to the side. Figure 8 shows a bird’s-eye view of the camera geometry in the object moving scenario. The distance between *x*_1_ and *x*_2_ can be calculated using the similarity of triangles. As Δ*PAO*_2_ and Δ*Px*_1_*x*_2_ are similar, Equation (8) is established.

(8) $$\frac{c}{p\left(x_{2}-x_{1}\right)}=\frac{h}{x}\;x_{2}=x_{1}+\frac{cx}{ph}$$

The length of $\bar{AO_{2}}$, *x*, can be defined by Equation (9) based on the triangle Δ*AO*_1_*O*_2_. Therefore, in the same way, the RSE and the vertical distance *h* can be calculated using Equation (10). Meanwhile, the distance between the camera and the object can be expressed as Equation (11)

(9) $$\tan\alpha =\frac{x}{v\Delta t}\;x=v\Delta t\tan\alpha$$

(10) $$\begin{array}{c} \tan\theta =\frac{x_{2}-x_{1}}{y_{2}-y_{1}}=\frac{\frac{cv\Delta t\tan\alpha }{ph}}{\frac{n\Delta t}{s}}=\frac{cvs\tan\alpha }{nph}\\ =\frac{\left(focal\;length\right)\times \left(velocity\right)\times \left(shutter\;speed\right)\times \tan\alpha }{\left(number\;of\;rows\right)\times \left(pixel\;size\right)\times \left(vertical\;distance\right)} \end{array}$$

$$vertical\;distance=\frac{\left(focal\;length\right)\times \left(velocity\right)\times \left(shutter\;speed\right)\times \tan\alpha }{\left(number\;of\;rows\right)\times \left(pixel\;size\right)\times \left(\tan\theta \right)}$$

(11) $$\begin{array}{ll} distance & =\frac{vertical\;distance}{\cos\alpha }\\ & =\frac{vertical\;distance}{\cos\left[\left(\arctan\left(\frac{\left|\left(x_{1}-p_{x}\right)p\right|}{c}\right)\right)\right]}\\ & =\frac{\left(focal\;length\right)\times \left(velocity\right)\times \left(shutter\;speed\right)\times \tan\alpha }{\left(number\;of\;rows\right)\times \left(pixel\;size\right)\times \tan\theta \times \cos\left[\left(\arctan\left(\frac{\left|\left(x_{1}-p_{x}\right)p\right|}{c}\right)\right)\right]} \end{array}$$

**Figure 9:**
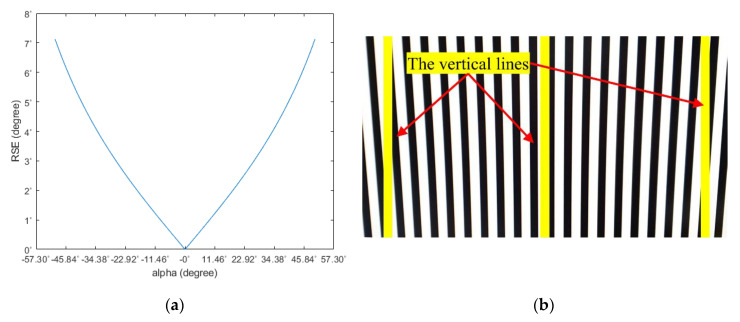
RSE occurrence test result: (**a**) simulation data using arbitrarily set parameters and (**b**) the image taken when the camera movement is perpendicular to the image plane.

From Equation (10), when the camera moves perpendicular to the object plane, the amount of RSE increases as the horizontal projection angle α between the center of the camera and object increases. Figure 9a is the simulation result of RSE as α changes. Figure 9b is the RSE acquired from the indoor test. As shown in Figure 9b, RSE equals zero and increases from the center to the boundary of the test image.

#### 2.2.3. Derivation of the RSE Equation: The Movement of the Object has a Certain Angle to the Image Plane

**Figure 10:**
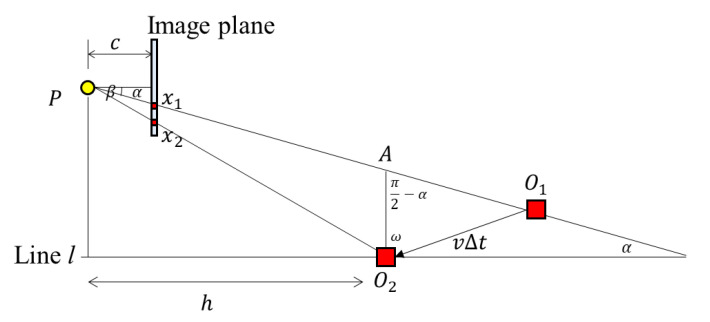
The RSE occurrence with the camera parameters and the moving scenario parameters (object moves diagonally).

Similar to other cases, *y*_2_ − *y*_1_ is derived as Equation (1), while *x*_2_ − *x*_1_ can be derived using the following Figure 10. Figure 10 shows an object moving in the angular direction of *ω* on the image plane. By the law of sines, the length of $\bar{AO_{2}}$ can be derived by Equation (12).

(12) $$\frac{v\Delta t}{\sin\left(\frac{\pi }{2}-\alpha \right)}=\frac{x}{\sin\left(\frac{\pi }{2}-\left(\omega -\alpha \right)\right)}\;\frac{v\Delta t}{\cos\alpha }=\frac{x}{\cos\left(\omega -\alpha \right)}\;x=\frac{v\Delta t\cos\left(\omega -\alpha \right)}{\cos\alpha }$$

*x*_2_ − *x*_1_ can be derived in the same way as in Equation (8). Therefore, the relationship between the RSE and the parameters is given by Equation (13). The distance between the camera and the object can be expressed as Equation (14).

(13) $$\begin{array}{c} \tan\theta =\frac{x_{2}-x_{1}}{y_{2}-y_{1}}=\frac{\frac{cv\Delta t\cos\left(\omega -\alpha \right)}{ph\cos\alpha }}{\frac{n\Delta t}{s}}=\frac{cvs\cos\left(\omega -\alpha \right)}{nph\cos\alpha }\\ =\frac{\left(focal\;length\right)\times \left(velocity\right)\times \left(shutter\;speed\right)\times \cos\left(\omega -\alpha \right)}{\left(number\;of\;rows\right)\times \left(pixel\;size\right)\times \left(vertical\;distance\right)\cos\alpha } \end{array}$$

$$vertical\;distance=\frac{\left(focal\;length\right)\times \left(velocity\right)\times \left(shutter\;speed\right)\times \cos\left(\omega -\alpha \right)}{\left(number\;of\;rows\right)\times \left(pixel\;size\right)\times \left(\tan\theta \right)\times \cos\alpha }$$

(14) $$\begin{array}{ll} distance & =\frac{vertical\;distance}{\cos\alpha }\\ & =\frac{vertical\;distance}{\cos\left[\left(\arctan\left(\frac{\left|\left(x_{1}-p_{x}\right)p\right|}{c}\right)\right)\right]}\\ & =\frac{\left(focal\;length\right)\times \left(velocity\right)\times \left(shutter\;speed\right)\times \cos\left(\omega -\alpha \right)}{\left(number\;of\;rows\right)\times \left(pixel\;size\right)\times \tan\theta \times \cos\alpha \times \cos\left[\left(\arctan\left(\frac{\left|\left(x_{1}-p_{x}\right)p\right|}{c}\right)\right)\right]} \end{array}$$

**Figure 11:**
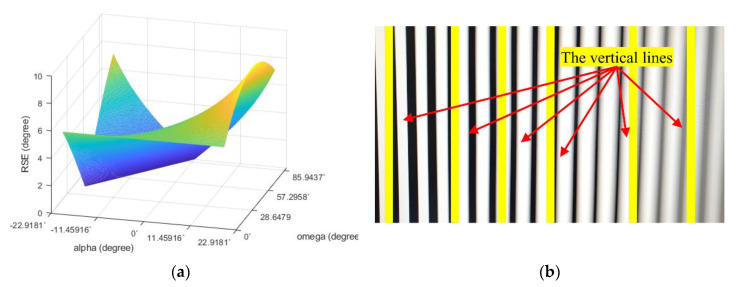
RSE occurrence test result: (**a**) simulation data using arbitrarily set parameters and (**b**) the image taken when the movement of the camera has a certain angle to the image plane.

To check the pattern of the RSE when the camera moves at an arbitrary angle to the object plane, the comparison of simulation and the indoor test was done. Figure 11a is the result of the simulation as the angle alpha and omega changes. Figure 11b is the image of RSE from the indoor test. The angle α in Figure 11a is the same as in Equation (10) and the angle ω is an inclined angle between the image and object plane. The test result exhibited similar patterns as Figure 9b but RSE was not zero any more at the center of the test image.

## 3. Experimental Result and Discussion

### 3.1. Sensors and Simulation Result

In this study, we checked the RSE occurrence with a Nikon D750, a DSLR camera frequently used in MMS, and an AF-S Nikkor 50 mm f/1.8G lens. This camera was selected because it is a full-frame rolling shutter type [26], and a single lens was selected to prevent changes in the camera parameters due to changes in the focal length [27]. Table 1 presents the specifications and calibration results of the camera. The distortions of the photographs were removed using the calibration results.

**Figure 12:**
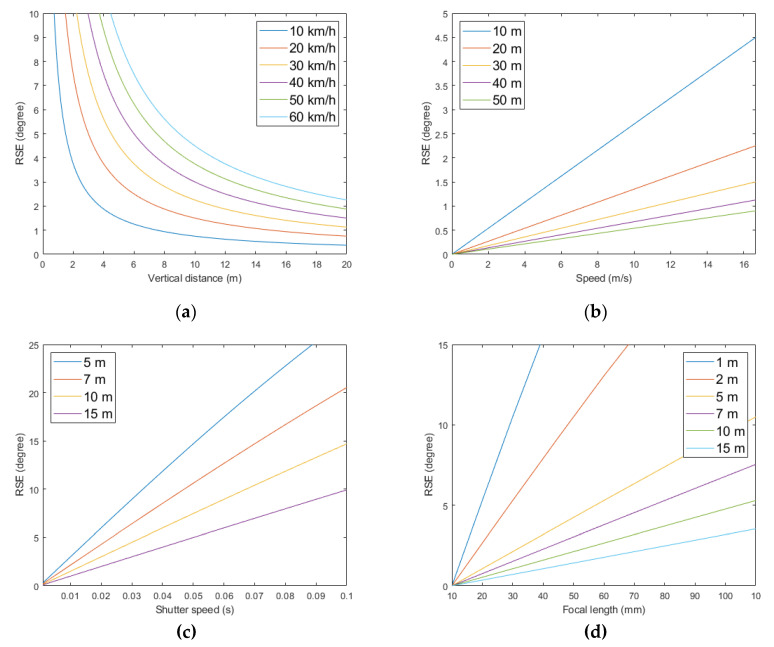
The RSE occurrence graph with respect to the parameters: (**a**) vertical distance-RSE; (**b**) speed-RSE; (**c**) shutter speed-RSE; and (**d**) focal length-RSE.

Figure 12 shows the simulation results of the RSE using the D750 with a 50 mm lens. Figure 9a,b shows the relationships of the RSE with the vertical distance and the speed, respectively, when the shutter speed was fixed at 1/50. Figure 12c is a graph showing the change in RSE when changing the shutter speed under the same conditions, and Figure 12d is the graph when changing the focal length. In Figure 12a,b, it can be seen that the RSE was inversely proportional to the vertical distance and had a linear relationship with the speed. In particular, as the vertical distance approached 0, the size of the RSE increased rapidly to close to 90°. As the distance between the camera and the object increased, the change in the RSE became smaller. In Figure 12c,d, it can be seen that the RSE also had a linear relationship with shutter speed and focal length. However, in Figure 12d, it can be predicted that the use of a lens with a short focal length (wide-angle lens) makes it difficult to observe the RSE of the distant object.

### 3.2. Field Test

**Figure 13:**
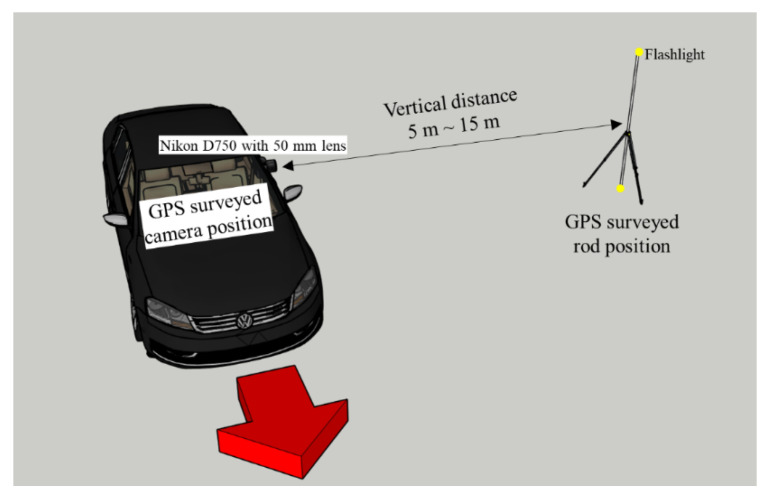
Illustration of the RSE experiment.

In this section, we investigated the usability of the RSE equation when dealing with field data. For the field test, a vertical 2-m-high rod was photographed using a DSLR camera with a 50 mm lens on a mobile platform. Flashlights were placed at the top and bottom of the rod to measure the exact angle of the RSE. In addition, to calculate the precise distance, the installation position of the rod and the position of the camera at the time of photographing were surveyed using GPS. Figure 13 shows an illustration of the experiment. The shutter and aperture values remained constant, with 1/50 s and f/16 for the deep depth of field (all areas of the photo are in focus), respectively. The focus was set to manual focus, and the focus distance was set to infinity.

**Figure 14:**
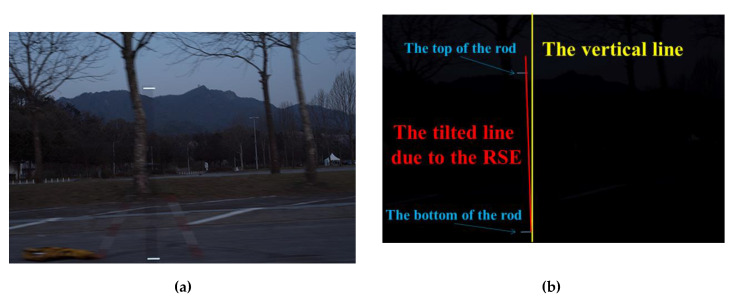
RSE observation: (**a**) brightened image; (**b**) original image with the RSE observation.

The experiments conducted in this study were divided into eight cases. In cases 1–3, the vertical distance was approximately 5 m, the speed was increased from 8.33 (30 km/h) to 13.89 m/s (50 km/h) at 2.78 m/s (10 km/h) intervals. In cases 4–6, the speed was 8.33 m/s while the vertical distance was changed to approximately 7, 10, and 15 m. Not only with varying distance and speed but also with varying shutter speed, the experiment was performed to check the effect of changes in camera parameters on RSE. Case 7 shows the results at shutter speed 1/25 s and Case 8 at shutter speed 1/60 s. In both cases, the vertical distance was 5 m, and the moving speed was 40 km/h. All distances and velocities were measured using GPS. The RSEs were measured five times in each case. Figure 14 shows a sample image of the RSE occurrence. Figure 14a shows the photo as bright as possible, and Figure 14b shows the RSE. The conditions of the photo were made to ease checking the RSE by reducing the ISO (mean film sensitivity) of the image as much as possible and closing the aperture as much as possible (f/16).

**Figure 15:**
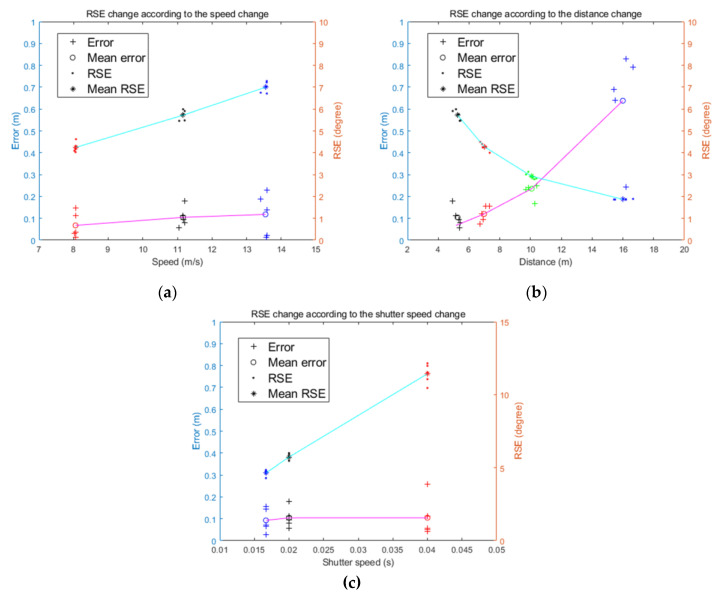
Field test results: (**a**) RSE and error change according to the speed change; (**b**) RSE and error change according to the distance change; and (**c**) RSE and error change according to the shutter speed change.

Table 2 and Figure 15 show the test results. Figure 15a–c shows the change of the RSE according to the speed, distance, and shutter speed changes, respectively. As shown in Figure 12 and Figure 15, it can be seen that the RSE changed linearly with the speed change and inversely with the distance change. However, the distance error between the camera and the object estimated using the RSE showed different patterns. While changing the speed of the camera platform, the distance error was maintained on average less than 0.2 m, but as the distance increased between the camera and the object, the distance error increased dramatically. This is because the RSE change becomes more sensitive to the distance change as the distance between the object and the camera increases. According to Equation (7) and Figure 12, when the camera speed was 40 km/h, the RSE change was 2.987° for 5–10 m, 1.000° for 10–15 m, and 0.500° for 15–20 m. This indicates that as the distance increases, the error of the RSE angle measurement can greatly influence the quality of the distance measurement result. Moreover, as the measurement of the RSE was performed using pixel units, the difficulty of precisely measuring the RSE affected the error of the distance measurement. As Δx and Δy in the near-field RSE were relatively large, the angle could be accurately measured. However, when measured from a long distance, the extent of the RSE was small, and the sizes of Δx and Δy were measured because the object was photographed in small size due to perspective. In the cases of 5, 7, and 10 m, the degrees of the RSE were measured differently according to a small change in distance. However, in the case of 15 m, it can be seen that the RSE was almost similar although the distance was slightly different. This may be because the extent of the RSE was similar, but it can also be considered that the RSE is the object of being measured and not the person that is doing the measurement. Therefore, increasing the measurement precision of the RSE can reduce the distance estimation error, and increasing the sensor resolution and using an image processing technique such as the RSE measurement in subpixel units enables a precise RSE measurement.

In Figure 15c, it was confirmed that the size of the RSE linearly increased as the shutter speed became slower (as the second increased), similar to the simulation results in Figure 12c. Additionally, in Figure 12c and Figure 15c, the errors of distance estimation with changes in shutter speed remained constant.

The results obtained in this study were compared with the results of low-cost LiDAR. Konolige et al. [28] measured the accuracy of a low-cost laser sensor. As a result, a 3 cm accuracy was secured at a 6 m distance (0.005 m error per distance), and this accuracy was reported to increase as a function of 1/x as the distance between the sensor and the object increased. In particular, it showed high measurement accuracy at a distance of 1 m or less but showed low performance when the distance increased to 6 m or more. In a study by Chen et al. [29], an ultra-tiny laser range sensor was developed to evaluate the accuracy. The sensor showed a standard deviation of 1.959 cm at 1.254 m. In comparison, the methodology proposed in our study shows lower accuracy than that of small laser equipment near the 6 m range, but compatible with low-cost laser in the longer range of 6 m.

The distance between the object and the camera could be estimated using a single photograph taken with a rolling-shutter-type camera as shown in the study. To obtain the depth between the object and the camera, an efficient and effective method can be used without the need for equipment, such as a LiDAR, depth camera, or stereo image. This study is useful for indoor mapping, robotics, MMS, etc. However, to actualize this study, increasing the accuracy of the distance measurement is required. For this, it is necessary to calibrate the operating mechanism of the rolling shutter, and through various attempts, knowledge of the optimal parameters, like the camera sensor resolution, shutter speed, focal length, and moving speed, must be accumulated. Furthermore, it is necessary to discuss the removal of blur caused by movement and to measure an accurate RSE angle. In this experiment, the RSE angle was relatively accurately measured by darkening the image and hanging flashlights at both ends of the vertical rod. However, as special equipment cannot be installed under normal circumstances, an image processing technique that can accurately measure the RSE must be developed. The development of precision measurement technology will increase the application distance range of the algorithm proposed in this paper, so it can be used in a wider range of fields.

## 4. Conclusions

We conducted a study to estimate the distance between a camera and an object using a single photo taken with a rolling-shutter-type camera. Based on the principle of the rolling shutter camera, an equation to estimate the distance was derived using the angle of the RSE and the camera parameters. The equation was verified by changing the distance between the object and the camera and the speed of the camera. When the camera moved at a speed of 30 km/h or more, the estimated distance had an error of approximately 10 cm regardless of the speed change. When the distance between the object and the camera was within 7 m, it was possible to estimate the distance within 10 cm, but the estimated distance error increased as the distance increased. Even though the shutter speed was changed, the distance could be estimated with an accuracy of less than 10 cm. Through theoretical analysis and experiment, it was shown that the distance can be estimated using a single photo taken using a rolling shutter camera if the appropriate parameters (sufficient speed of the camera or object, close range, adjustment of shutter speed, etc.) are controlled. To use the proposed method for practical engineering applications, an automatic selection of appropriate parameters among camera settings is required. It is also required to develop image processing techniques that can accurately measure the amount of RSE. The suggested equations can be readily used to complement or replace expensive LiDAR or radar devices used for indoor mapping, robotics, and autonomous driving.

## Figures and Tables

**Table 1:** Specifications and calibration results of the camera.

Nikon D750 with AF-S Nikkor 50 mm f/1.8 G (Video Mode)		
Specifications		
Sensor Size		35.9× 24 mm
Image resolution		6010 × 4010
Video resolution		1920 × 1080
Pixel size		5.98 μm
**Parameters**		**Value**
Focal length	*f_cx_*	54.230267 mm
	*f_cy_*	54.448138 mm
Principal point	*pc_x_*	3132.23992 pixels
	*pc_y_*	1706.34214 pixels
Skew		0.0000
Radial distortion parameters		[−0.12818 1.21296]
Tangential distortion parameters		[−0.00898 0.00315]

**Table 2:** RSE test results.

Case	Speed (m/s)	Distance (m)	RSE (°)	Estimated Distance (m)	Error (m)
Case 1 (30 km/h, 5 m)	8.0591	5.2054	4.17	5.2191	0.0137
	8.0564	5.4532	4.03	5.4179	0.0353
	8.0676	4.8304	4.62	4.7169	0.1135
	8.0637	4.9168	4.30	5.0627	0.1459
	8.0221	5.3246	4.09	5.2952	0.0294
Case 2 (40 km/h, 5 m)	11.2101	5.4408	5.48	5.5200	0.0792
	11.2115	4.9365	5.91	5.1153	0.1788
	11.1760	5.3313	5.76	5.2365	0.0948
	11.1636	5.1477	5.99	5.0356	0.1121
	11.0520	5.3982	5.46	5.4553	0.0571
Case 3 (50 km/h, 5 m)	13.5850	4.9982	7.28	5.0206	0.0224
	13.5862	5.2270	6.71	5.4563	0.2293
	13.5744	5.1882	7.03	5.2008	0.0126
	13.5815	5.2039	7.22	5.0662	0.1377
	13.4091	5.1584	6.75	5.3463	0.1879
Case 4 (40 km/h, 7 m)	10.8343	6.8436	4.40	6.9635	0.1199
	11.0844	6.7237	4.50	6.6481	0.0756
	11.0032	6.8947	4.25	6.9898	0.0951
	11.1035	7.3434	4.00	7.4994	0.1560
	10.9582	7.0984	4.26	6.9435	0.1549
Case 5 (40 km/h, 10 m)	10.9093	9.703	3.01	9.9355	0.2325
	10.7693	10.2543	2.79	10.4228	0.1685
	11.1222	10.3869	2.83	10.6349	0.248
	11.1384	9.8542	3.13	9.6141	0.2401
	11.0771	10.1456	2.87	10.4352	0.2896
Case 6 (40 km/h, 15 m)	11.0834	15.4933	1.86	16.1324	0.6391
	11.0470	16.6647	1.89	15.8734	0.7913
	11.0691	15.4279	1.86	16.1166	0.6887
	11.0768	16.2025	1.85	15.3739	0.8286
	11.0527	16.1976	1.88	15.9551	0.2425
Case 7 (shutter speed 1/25 s)	11.0357	5.2785	11.06	5.3222	0.0437
	10.8491	5.5469	10.46	5.6047	0.0578
	10.9340	4.8003	12.15	4.7480	0.0523
	11.1712	4.9649	11.98	5.2230	0.2581
	11.0971	5.1576	11.52	5.2688	0.1112
Case 8 (shutter speed 1/60 s)	11.3816	5.6948	4.61	5.5492	0.1456
	11.0655	5.3171	4.74	5.2525	0.0646
	11.1952	5.9502	4.29	5.8793	0.0709
	11.1869	5.0344	4.85	5.1891	0.1547
	11.4898	5.4387	4.73	5.4663	0.0276
